# Plant genetic variation drives geographic differences in atmosphere–plant–ecosystem feedbacks

**DOI:** 10.1002/pei3.10031

**Published:** 2020-09-28

**Authors:** Shannon L. J. Bayliss, Liam O. Mueller, Ian M. Ware, Jennifer A. Schweitzer, Joseph K. Bailey

**Affiliations:** ^1^ Department of Ecology and Evolutionary Biology University of Tennessee Knoxville TN USA; ^2^ USDA Forest Service Pacific Southwest Research Station Institute of Pacific Islands Forestry Hilo HI USA

**Keywords:** biomass, budyko, earth–atmosphere feedback, eco‐evo feedback, energy, stoma, water

## Abstract

The objective of this study was to understand how genetic variation in a riparian species, *Populus angustifolia*, affects mass and energy exchange between the land and atmosphere across ~1,700 km of latitude of the western United States. To examine the potential for large‐scale land–atmosphere feedbacks in hydrologic processes driven by geographic differences in plant population traits, we use a physical hydrology model, paired field, and greenhouse observations of plant traits, and stable isotope compositions of soil, stem, and leaf water of *P. angustifolia* populations. Populations show patterns of local adaptation in traits related to landscape hydrologic functioning—a 47% difference in stomatal density in greenhouse conditions and a 74% difference in stomatal ratio in the field. Trait and stable isotope differences reveal that populations use water differently which is related to historical landscape hydrologic functioning (evapotranspiration and streamflow). Overall, results suggest that populations from landscapes with different hydrologic histories will differ in their ability to maintain favorable water balance with changing atmospheric demands for water, with ecosystem consequences.

## 1 INTRODUCTION

Alterations to species distributions will accompany global climatic changes, consequently destabilizing the functions and services that diverse ecosystems provide (Burrows et al., 2011; Naeem, Duffy, & Zavaleta, 2012; Parmesan, 2006; Urban, 2015). However, much of our understanding of distribution shifts are limited by the common assumption in models that all populations of a species will respond in a similar manner to environmental changes, despite knowledge to the contrary (Benito Garzón, Robson, & Hampe, 2019; Gotelli & Stanton‐Geddes, 2015; Peterson, Doak, & Morris, 2019). Furthermore, research to date tends to over‐simplify the relationship between temperature and precipitation on the landscape, rarely considering energy and water as a more dynamic relationship (Bates, Kundzewicz, Wu, & Palutikof, 2008; Jones et al., 2012). This interaction affects the atmospheric supply and demand of water that drives population, community, and ecosystem dynamics (Bates et al., 2008; Jones et al., 2012). Taken together, both water‐energy interactions and intraspecific variation in plant climatic tolerance vary across small and large spatial scales needs to be considered in order to understand feedbacks between population structure and large‐scale ecosystem processes (e.g., water fluxes; Bates et al., 2008; Hendry, 2017; Jones et al., 2012; Thompson, 2005).

The Budyko water‐budget model is widespread in the field of hydrology. The model considers the mass balance (amount of water) and energy balance (phase change potential) of systems by reflecting actual evapotranspiration (AET) as a function of precipitation (P) and potential evapotranspiration (PET; Budyko, 1974; Sposito, 2017). The theoretical model is useful for predicting water cycling in various climates, although values based on data (actual values) deviate from the theoretical curve to represent interactions with ecological factors such as soil type, vegetation cover, and/or other biotic factors (Gentine, D'Odorico, Lintner, Sivandran, & Salvucci, 2012; Troch, Carrillo, Sivapalan, Wagener, & Sawicz, 2013). While distributions of terrestrial species are most often described and strongly driven by patterns in soils, precipitation, temperature, and distance to water (Bradie & Leung, 2017), PET actually explains more variation in natural selection globally in terrestrial biomes than does temperature (Siepielski et al., 2017), and leaf economic traits are more strongly correlated with vapor pressure deficit and PET than it is with precipitation or temperature (Wright et al., 2004). Although PET is derived from temperature, it more accurately reflects the temperature during the period of time when plants are actively using energy and water (i.e., the growing season) (Eller et al., 2018; Siepielski et al., 2017).

As water and energy change in availability and variability on the landscape, plant population responses will likely vary due to differing amounts of intraspecific variation, genetic architecture or due to adaptations to differing historical abiotic conditions. For instance, the ability of plants to use water to produce biomass depends strongly on soil water availability which varies significantly across the landscape and is also affected by temperature (Beier et al., 2012; Rodriguez‐Iturbe & Porporato, 2005). If atmospheric demand for water increases (i.e., high atmospheric vapor pressure deficit), which is predicted to occur globally, plants must prevent excessive water loss (Grossiord et al., 2020). Rapid responses to high vapor pressure deficits include adjusting stomatal aperture, while longer‐term responses include altering the density, distribution, and size of stomatal pores (Bertolino, Caine, & Gray, 2019; Cowan & Farquhar, 1977; Hetherington & Woodward, 2003; McAdam & Brodribb, 2014; Oren et al., 1999). Genetically based variation stomatal density or size (Mitton, Grant, & Yoshino, 1998) result in variations of maximum stomatal conductance, affect a plant's ability to manage limited resources, and affect large‐scale ecosystem processes (Novick et al., 2016). Transpiration directly supports primary productivity, biomass accumulation, and carbon assimilation, thus is directly related to carbon, water, and energy fluxes on the landscape (Hetherington & Woodward, 2003; Kominoski et al., 2013; Sposito, 2017). Here, we consider variation in historic water cycling on the landscape and examine local adaptation of plant populations to understand ecological and evolutionary linkages on a landscape scale.

Using the Budyko physical hydrology model, paired field and greenhouse observations of *P. angustifolia* traits, and stable isotope compositions of soil, stem, and leaf water, this study considers the potential for large‐scale land–atmosphere feedbacks in hydrologic processes driven by geographic differences in plant population traits. With the observation that the supply and atmospheric demand for water, as well as water use differ on the landscape across populations of *P. angustifolia*, we test the following specific hypotheses: (a) Populations of *P. angustifolia* show genetic divergence in stomatal density, stomatal distribution, stomatal size, and aboveground biomass, (b) consistent with patterns of genetic divergence and local adaptation, stomatal traits are related to hydrologic variables on the landscape, (c) populations draw water from different sources (e.g., stream water or precipitation), and (d) populations vary in water use given atmospheric demands. Overall, results show that divergent plant populations have evolved in response to geographic variation in dryness.

## 2 MATERIALS AND METHODS

### 2.1 Building site‐level energy and water budgets

**FIGURE 1:**
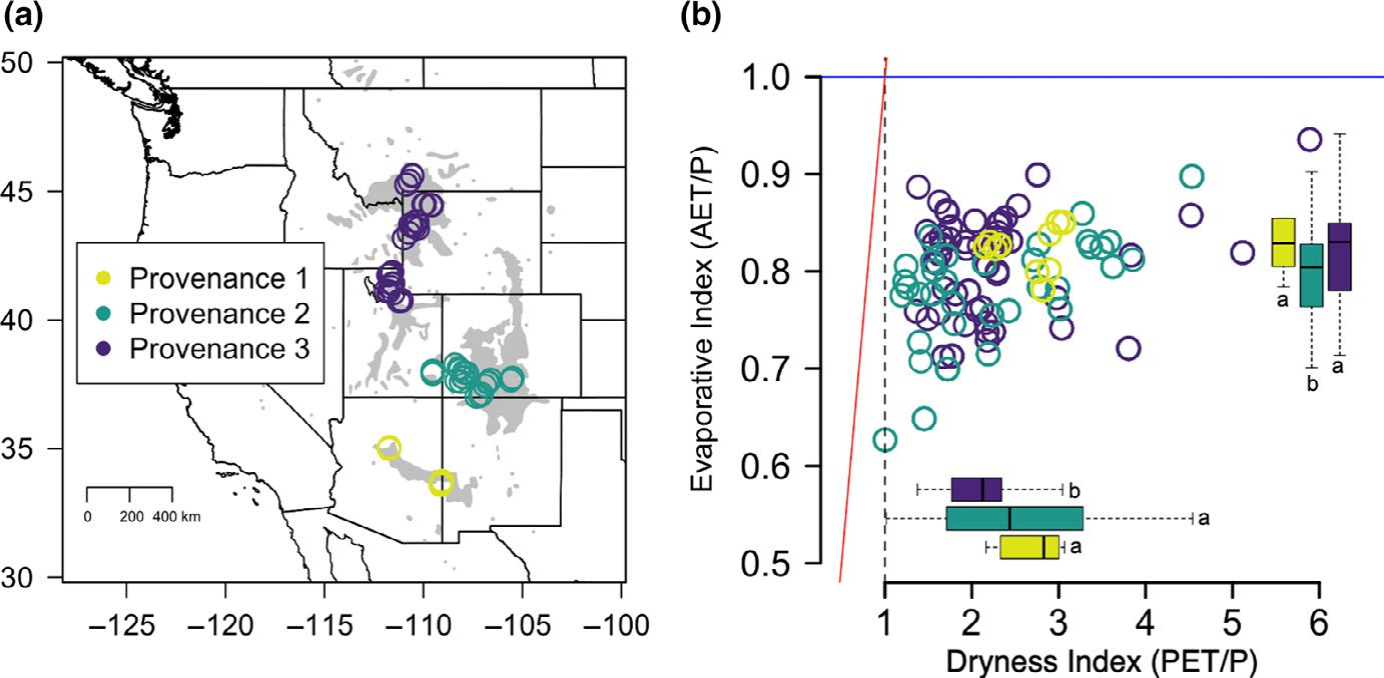
(a) Map of field collection sites, genetic provenances, and geographic range of *Populus angustifolia*. Each point represents a unique sampling location: there are multiple collection sites within each of the 17 populations of the three genetic provenances (provenance represented by color). (b) Field collection sites within the 17 *Populus angustifolia* populations plotted with Budyko Model Parameters. AET = Actual Evapotranspiration; P = Precipitation; PET = Potential Evapotranspiration. The blue line represents a water limit (AET = P), at which 100% of water supplied to the landscape as precipitation (P) is cycled back to the atmosphere through evapotranspiration (AET). The red line represents an energy limit (AET = PET), at which the amount of water recycled to the atmosphere through evapotranspiration (AET) meets the atmospheric demand for water (PET). Genetic provenance is represented by color as in panel A. Points are plotted using observed values of mean annual P, PET, and AET from georeferenced locations of field collection sites. Inset boxplots show provenance differences in the two Budyko parameters, with letters referring to statistically significant differences between provenances from post‐hoc Tukey Contrasts

We built energy and water budgets using Budyko model parameters describing how precipitation (P) is recycled to the atmosphere via actual evapotranspiration (AET) or held on land as streamflow (Q) across a continuum of humid to arid systems (PET/P; Figure 1b; Budyko, 1974). The theoretical model (note, the modeled curve is not depicted in Figure 1b) provides expectations for the energy balance and water use based on physical processes (i.e., evapotranspiration consumes heat as latent energy flux during the phase change of liquid water to vapor) and the assumption that P = Q + AET (Budyko, 1974; Trenberth, Fasullo, & Kiehl, 2009; Wang & Dickinson, 2012). Values derived from real data represent long‐term patterns describing how water *actually* cycles on the landscape, taking into account more than physical processes—in other words, landscape variation in interactions between soil, vegetation, and atmospheric conditions. The dryness index (PET/P) on the x‐axis of the model represents the aridity of the climate, with values greater than 1 indicating arid climates whereby plants are limited by water rather than by energy. The evaporative index (AET/P) on the y‐axis describes how precipitation is distributed on land, or the percentage of P recycled back to the atmosphere through AET. An energy limit exists where AET = PET (i.e., demand limit; at which atmospheric demand for water is met), and a mass limit exists where AET = P (also known as a water limit, or supply limit; i.e., 100% of P is partitioned back to the atmosphere) (Budyko, 1974; Creed et al., 2014; Jones et al., 2012). In this paper, we are explicitly interested in unique long‐term patterns of water cycling on the landscape which capture landscape heterogeneity, and not in the theoretical predictions (Figure 1b; Gentine et al., 2012; Troch et al., 2013). We extracted mean annual precipitation from WorldClim (Fick & Hijmans, 2017), and mean annual PET and AET from the CGIAR‐CSI GeoPortal (Trabucco & Zomer, 2009) using geo‐referenced locations of our collection sites.

### 2.2 Study species and sites

To understand plant–energy–water relationships we used a dominant riparian tree species: *Populus angustifolia* James (Rood, Nielsen, Shenton, Gill, & Letts, 2010), the narrowleaf cottonwood, that is widely distributed along the Rocky Mountains from northern Mexico to southern Canada (Evans et al., 2015) and span large precipitation, temperature, stream flow, and soil water gradients. Cottonwoods, *Populus ssp*., are an ideal study system for examining these relationships as they show intraspecific variation in physiological and morphological responses to changes in the water cycle, including groundwater, precipitation, and streamflow (Rood, Braatne, & Hughes, 2003). Furthermore, *Populus ssp*. are foundation species in riparian ecosystems in the western U.S. contributing greatly to ecosystem transpiration, but have been generally labeled as “drought sensitive” species that are declining in recent years (Kominoski et al., 2013; Schaeffer, Williams, & Goodrich, 2000). It is also clear that these riparian forests do not receive enough precipitation during the growing season to support the levels of transpiration to meet atmospheric demand (Flanagan, Orchard, Logie, Coburn, & Rood, 2017; Scott, Shuttleworth, Goodrich, & Maddock III, 2000; Yang, Rood, & Flanagan, 2019). Populations under such environmental constraints are ideal for identifying genetic divergence in response to varying hydrologic dynamics.

We have established field sites along 17 rivers in the western United States that span significant environmental gradients and nearly 1,700 kilometers of latitude (Figure 1a). In 2012, over 525 genotypes of *P. angustifolia* were collected and geo‐located from multiple (minimum three, maximum five) sites along each river, including at the highest and lowest elevations. The collected trees have been established in a greenhouse at the University of Tennessee and all tree replicates were tagged with a number and randomized in the common environment to minimize microspatial variation in light or temperature (details in Ware, Van Nuland, et al., 2019). This is a conservative experimental approach to examining genetic variation at multiple genetic hierarchies, including provenance, population, site, and genotype, that reduces observer sample bias. No plants were water limited in the greenhouse, and temperature conditions were maintained between 65 and 75 degrees Fahrenheit. Testing for variation in trait measurements in the common environment and relating these traits to environmental parameters allows us to infer patterns of local adaptation (Kawecki & Ebert, 2004; Leimu & Fischer, 2008). We refer to populations as groupings of all genotypes from sites along each river, resulting in 17 river populations. These 17 populations vary locally and regionally, grouping into three genetically distinct provenances which have been geographically isolated by large landscape features including the Great Basin, the Rocky Mountains, and the Mogollon Rim (Figure 1a; Evans et al., 2015).

### 2.3 Plant functional and performance traits

Field biomass measurements were made in the summer of 2012, and greenhouse biomass measurements were later made from established clonal cuttings of the same genotypes in 2016. In June 2017, we re‐visited a subsample of the genotypes visited in 2012 to obtain field stomatal measurements. At this time, we also collected cuttings and established clones in the greenhouse. We measured the same suite of stomatal traits on these trees between October 27 and 31, 2017. Details of trait measurements are described below. Because development and leaf age can affect stomatal traits (e.g., Hamanishi, Thomas, & Campbell, 2012; Pearce, Millard, Bray, & Rood, 2005), we checked for ontogenetic differences in traits between older clones from which biomass was derived and their respective younger clones (from the same “source” tree in the field). Seven of the same genotypes were measured for stomatal traits in the “older” (2012) trees in October 2017. A two‐tailed unpaired *t* test on stomatal density and stomatal distribution showed no difference between the two age groups (*p = .56*,*p = .39*, respectively).

#### 2.3.1 Aboveground biomass

In the field, aboveground biomass estimates of *P. angustifolia* genotypes were made in 2012 by measuring tree circumference (m) which was used to calculate DBH (cm): DBH = 100 × circumference/3.14). We estimated biomass (kg) using an allometric equation for *Populus* from Chojnacky et al. (2014) who developed from a meta‐analysis of 10 existing allometric equations based on tree DBH: Aboveground biomass (kg) = −2.6863 + ((2.4561) × ln(DBH)). In the greenhouse, aboveground biomass measurements were made in 2016, 4 years after cuttings were established in the common environment. To estimate biomass (grams of C) for saplings, we created an allometric equation using six *P. angustifolia* genotypes grown in the greenhouse environment and measurements collected across 3 years (June 2012, 2013, and 2014; described and used in Van Nuland et al. (2017) and Ware et al. (2019, 2019)). The following allometric equation was used: Aboveground biomass (g) = (stem volume (mm^3^) × 0.41899) − 2.40137.

#### 2.3.2 Stomatal traits

We measured three traits related to stomatal function: density; distribution, and size. Stomata control the movement of gases in and out of the leaf (e.g., carbon dioxide for photosynthesis, water via transpiration). Variation in the size and the density of stomata as well as the location on leaf surfaces (i.e., adaxial (top), abaxial (bottom)) reflect ways that plants can control water loss, and thus are important to plant function (Aasamaa, Sõber, & Rahi, 2001; Bertolino et al., 2019; Cornelissen et al., 2003; Hetherington & Woodward, 2003; Sack, Melcher, Liu, Middleton, & Pardee, 2006). Prior studies on *Populus* reveal positive relationships between stomatal density and ratio with conductance and carbon assimilation rates (Guy & Gornall, 2007; Pearce et al., 2005; Soolanayakanahally, Guy, Silim, Drewes, & Schroeder, 2009) and changes in water‐use efficiency with drought (Hamanishi et al., 2012).

Leaves were collected in the field in June 2017 from two genotypes along (3 sites) six rivers distributed across the three genetic provenances (Provenance 1: Blue River, NM and Oak Creek, AZ; Provenance 2: San Miguel River, CO and Indian Creek, UT; Provenance 3: Weber River, UT and Snake River, WY). These collections resulted in six genotypes per river, or 12 genotypes per genetic provenance, and were the same genotypes that were visited in 2012 collection described above. We chose three leaves from the terminal shoots of lower exterior branches of each tree to minimize intra‐canopy and age variation in stomatal density (Sack et al., 2006). Impressions of the leaf epidermis were made on the adaxial and abaxial side of each leaf using clear nail varnish and tape, then individually arranged on glass slides. Counts were made in the software ImageJ (Schneider, Rasband, & Eliceiri, 2012) from light microscopy photographs with a 10X objective. We calculated the total number of stomata per area by adding the number of stomata on both leaf surfaces (henceforth “stomatal density”), and we calculated the relative placement of stomata by calculating the ratio of adaxial density to abaxial density (henceforth “stomatal ratio”). These methods resulted in six impressions per genotype, or 216 total impressions. Finally, we made 20 measurements of stomatal pore length on each photograph in ImageJ (Schneider et al., 2012). As stomatal density on photographs was often higher than 20, we overlayed a grid in ImageJ and randomly selected a row across which to begin measurements. If density was too low to obtain 20 measurements, more often on the adaxial impressions, we measured the pore length of every present stoma. These methods resulted in 120 pore length measurements per genotype. We repeated the same measures from leaves collected from the same genotypes of trees growing in the common environment, described above, although we lost three genotypes from the San Miguel and the Weber Rivers and one genotype from both the Blue River and the Snake River. Greenhouse measurements therefore consisted of a leaf collected from three clonal replicates of 28 genotypes.

#### 2.3.3 Water stable isotope measurements

We analyzed river water, stem, leaf, and soil samples for stable isotope measurements (δ^18^O and δ^2^H) to determine plant water source. In June 2017, at the mid‐elevation site along each of the six rivers, we collected stem and leaf samples for stable isotope analysis from two unique genotypes of *P. angustifolia*. Soil samples were collected from underneath each genotype at a depth of approximately 10 cm. River water samples were collected below the surface of the water in each of the six rivers. All samples were kept on dry ice until delivered to the Colorado Plateau Stable Isotope Laboratory (CPSIL; www.isotope.nau.edu) at Northern Arizona University in Flagstaff, AZ. The samples were stored in a freezer until extraction and analysis. Water was extracted from woody stem samples via cryogenic vacuum extraction. Samples were extracted in September, 2017 and analyzed for the stable oxygen (^18^O/^16^O) and hydrogen (^2^H/^1^H) ratios (expressed per mille). All of the extractions were made via LGR DLT‐100 laser spectroscopy.

### 2.4 Statistical Analyses

All analyses were performed using the statistical software R (version 3.6.1; R Development Core Team, 2016). To confirm our observations that water availability and the atmospheric demand for water vary across the range of *P. angustifolia*, we built linear models predicting variation in the two axes of the Budyko water budget (dryness index and evaporative index) with population. Separate models were built for the 17 populations examined for biomass, the six‐population subset used for stomatal measurements, and the three genetic provenances (with population as a random effect; R package lme4 (Bates et al., 2015)). Hypothesis testing for each linear model was done by marginal sums of squares ANOVA in the R package car (Fox et al., 2018) and the null hypothesis was rejected at an α = 0.05.

To test the hypothesis that stomatal and growth traits from *P. angustifolia* genetic provenances reveal patterns of local adaptation, we ran linear mixed effects models with biomass, stomatal density, and stomatal ratio as response variables, provenance as a fixed effect, and population (river) as a random effect in the lme4 R package (Bates et al., 2015). For stomatal density and ratio, genotype was also included as a random effect. Models were compared to null models with random effects only using likelihood ratio tests and by comparing AIC values. Post‐hoc pairwise differences comparisons were made of provenance‐level means with Tukey contrasts using the ghlt function in R package multcomp (Hothorn, Bretz, & Westfall, 2008) with the null hypothesis rejected at an α = 0.05.

To test the hypothesis that water‐regulation and functional traits are related to hydrologic variables on the landscape, we used restricted estimated maximum likelihood (REML) linear mixed models (R package lme4 (Bates et al., 2015)). We included water budget parameters as fixed effects and we included genetic provenance in models as a random effect to remove “blocked” variation that can be attributed to genetic grouping. Response variables included *P. angustifolia* greenhouse biomass, stomatal density, stomatal ratio, and stomatal pore length measurements. Hypothesis testing for each linear model was done by marginal sums of squares ANOVA in the car R package (Fox et al., 2018) and the null hypothesis was rejected at an α = 0.05.

To test the hypothesis that populations draw water from different sources, we used stable isotope values to calculate deuterium excess values (d‐excess) as d‐excess = δ^2^H – 8 × δ^18^O (Dansgaard, 1964). This metric represents deviations from the average global relationship between δ2H and δ18O in precipitation, the global meteoric water line (GMWL; Craig, 1961). Because the global relationship varies across latitudes and continents (for example; (Sprenger, Leistert, Gimbel, & Weiler, 2016), we also calculated local meteoric water lines (LMWL) that are regionally specific. We used precipitation isotopic signatures for the month of June (when samples were collected) obtained from the OIPC (The Online Isotopes in Precipitation Calculator; Bowen, 2019; Bowen, Wassenaar, & Hobson, 2005; Welker, 2000) by inputting the latitudes, longitudes, and elevations for sampling locations. From this, we derived line‐conditioned excess values (lc‐excess; Landwehr & Coplen, 2014; Sprenger et al., 2016), calculated as: lc‐excess = δ^2^H – a × δ^18^O – b, where a and b are the slope and intercept of the LMWL (Table 2, R2 = .988). Negative values of both d‐excess and lc‐excess represent water isotope ratios that have been evaporatively enriched.

## 3 RESULTS

### 3.1 Water supply (precipitation), atmospheric demand for water (potential evapotranspiration), and water use (actual evapotranspiration) differ across the range of *Populus angustifolia*

*Populus angustifolia* riparian forests across the western United States (Figure 1a **)** are water limited; all field sites fall to the right of 1 on the dryness index (where PET = P; Figure 1b), indicating that on average, all sites and populations are limited by the supply of water. Along this axis, however, sites span a large range of dryness (min = 1.00, max = 5.89; Figure 1b) and differ by genetic provenances (inset boxplots, Figure 1b). Additionally, all sites fall below 1 on the evaporative index (where AET = P; blue line, Figure 1b) indicating that no more water is recycled to the atmosphere than falls as precipitation. This is expected for annual averages, which are constrained by the amount of water available in a system. Site‐level values on this axis span from about 63% to 94% of water recycling to the atmosphere through AET annually (min = 0.63, max = 0.94) and differ across genetic provenances (inset boxplots, Figure 1b). Landscape differentiation in average dryness and evaporative indices across provenances is important, as is the situation of points within a single river on the plot, as this represents variation in water cycling regimes. For example, a river may span a wide range of climatic conditions (dryness index) but function similarly along points of the river (no variation in evaporative index, e.g. 80% of P goes to AET everywhere along the river), while another river may span a narrow range of climatic conditions (dryness index) but cycle water quite differently along the river (large range in evaporative index). In this way, the model parameters also capture effects of elevation. A comprehensive representation of sites from all 17 rivers can be found on the Budyko curve in the Figure S1a along with a representation of the six rivers visited for stomatal trait measurements (Figure S1b).

### 3.2 Populations of *P. angustifolia* show patterns of genetic divergence in traits related to the water cycle

**FIGURE 2:**
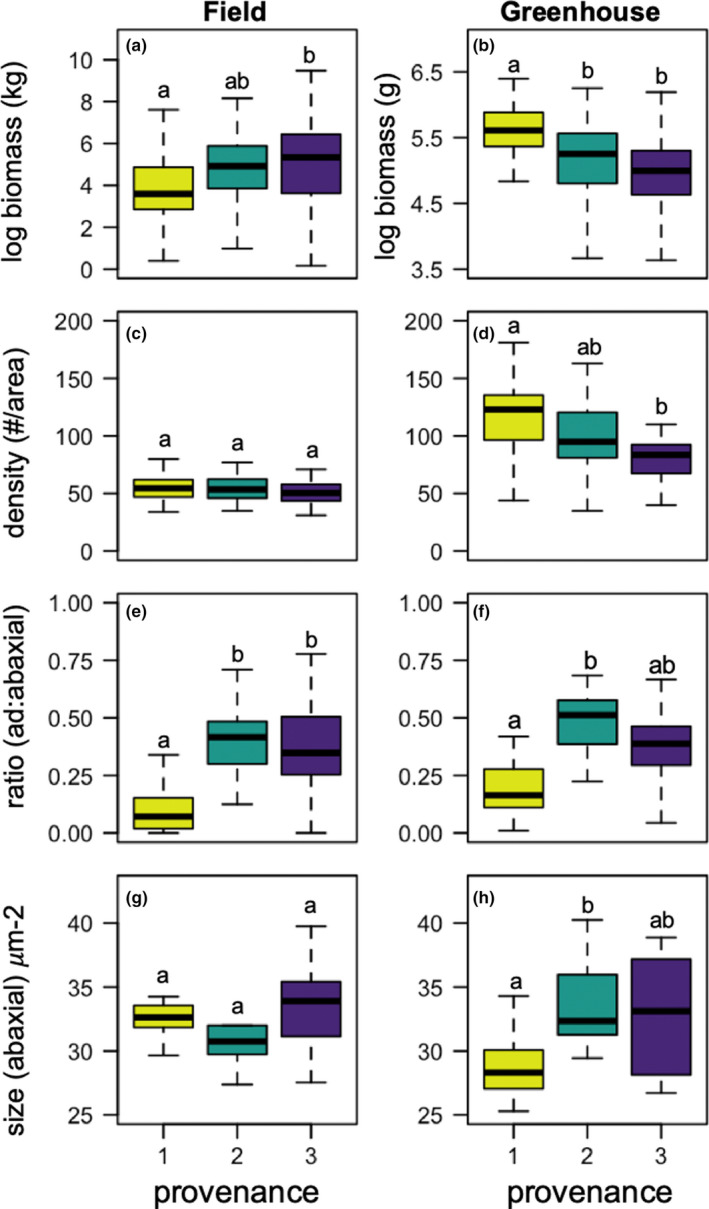
Genetic provenances of *Populus angustifolia* differ in traits relating to water‐use and ecosystem function. Letters refer to statistically significant differences between provenances from post‐hoc Tukey Contrasts. Note the difference in y‐axis scale between field and greenhouse biomass. (a) Field Biomass (kg), log transformed (Prov_3‐1_ *p* = .018); (b) Greenhouse Biomass (g), log transformed (Prov_2‐1_ *p* = .001; Prov_3‐1_ *p* < .001); (c) Field Stomatal Density (#/area); (d) Greenhouse Stomatal Density (#/area; Prov_3‐1_ *p* < .001); (e) Field Stomatal Ratio (ad:abaxial; Prov_2‐1_ *p* < .001; Prov_3‐1_ *p* < .001); (f) Greenhouse Stomatal Ratio (ad:abaxial; Prov_2‐1_ *p* = .068); (g) Field Abaxial Stomatal Pore Length (μm); (h) Greenhouse Abaxial Stomatal Pore Length (μm; Prov_2‐1_ *p* = .009; Prov_3‐1_ *p* = .059)

Biomass: In the field, we find that biomass is lowest in Provenance 1 compared to Provenances 2 and 3 which do not show significant differences (Figure 2a, Table 1 **)**. Conversely, in the common environment we find that Provenance 1 had the highest biomass (µ_p1_ = 294.8 g), while Provenance 3 had the lowest average aboveground biomass (µ_p3_ = 89.7 g). Overall, this represents a 69% genetically based difference in biomass across the three provenances. These results demonstrate a pattern of genetic divergence at the provenance level (Figure 2b, Table 1) and environmental constraints on biomass production in the field within the range of Provenance 1, likely related to limitations in the supply of water and plant strategies to mitigate water limitation. A post‐hoc Tukey test reveals significant differences between Provenances 1 and 2, and Provenances 1 and 3 (Table 1). Provenances 2 and 3 show marginally significant differences in biomass (*p* = .09; Table 1 **)** although these provenances have the lowest sample size (N_p2_ = 213 and N_p3_ = 75, respectively). To check if these differences could be explained by growth duration (e.g., Evans et al., 2016), we also ran models including growing season length in the greenhouse (recorded as the number of days between first bud break in the spring and plant senescence in the fall), and latitude as a proxy for growing season length in the field. Our findings did not change with consideration of these variables.

**TABLE 1:** Summary of the linear mixed effects model rankings for determining importance of provenance for biomass, stomatal density, distribution (ratio), and size (abaxial pore length) in the field [F] and in the greenhouse [GH]. River is included as random effect for all models. Genotype is also included as a random effect for stomatal models

Trait	Model rank	Main effects	AIC	*χ* ^2^, df	*p* (>Chisq)
[GH) Biomass	1	Provenance	4653.1	17.999, 2	**.000124**
	2	Null	4667.1		
[GH] Stomatal density	1	Provenance	785.90	10.04, 2	**.00660**
	2	Null	791.94		
[GH] Stomatal ratio	1	Provenance	−19.795	4.563, 2	.102
	2	Null	−19.233		
[GH) Abaxial pore length	1	Provenance	168.14	4.755, 2	.093
	2	Null	168.9		
[F] Biomass	1	Provenance	2140.8	7.273, 2	**.0263**
	2	Null	2144.1		
[F] Stomatal density	1	Provenance	874.97	1.646, 2	.439
	2	Null	872.62		
[F] Stomatal ratio	1	Provenance	138.44	12.502, 2	**.00193**
	2	Null	−129.94		
[F] Abaxial pore length	1	Provenance	161.16	7.181, 2	**.0280**
	2	Null	164.34		

Note

The bolded values are determined based on a significance threshold of *p* < .05.

Stomatal Traits: While the average stomatal density does not differ between provenances in the field (Figure 2c **;** Table 1 **)**, Provenance 1 shows 47.3% difference in stomatal density in the common environment compared to Provenance 3 (µ_p1_ = 118.1; µ_p3_ = 80.2; *p* < .001), and a 23.7% increase relative to Provenance 2 (µ_p2_ = 95.5; *p* = .062) (Figure 2d; Table 1 **)**. Overall, this represents nearly a doubling of the total number of stoma on leaf surfaces across the provenances. Additionally, we find that provenances differ in the field in stomatal ratio (Figure 2e; Table 1): Provenance 1 has a significantly lower stomatal ratio (µ_p1_ = 0.103) compared to Provenances 2 and 3 (which do not significantly differ from each other; µ_p2_ = 0.40 and µ_p3_ = 0.37, respectively). These data confirm those found previously, showing a species average of stomatal ratio to be about 0.32 (Pearce et al., 2005). In field conditions, Provenance 1 has more stomates on the abaxial leaf surface, and although the greenhouse trend reflects this field trends, the only emergent significant difference is between Provenances 1 and 2 (Figure 2f; Table 1). Finally, we found no significant differences between the three provenances in *adaxial* stomatal pore length. However, while *abaxial* stomatal pore length did not differ between provenances in the field (Figure 2g; Table 1), with an average length of 32.4µm, we did find differences in the greenhouse. Similar to the patterns of stomatal distribution in the greenhouse, abaxial stomatal pore length in the greenhouse of Provenance 1 (µ_p1_ = 29.0 µm) was significantly smaller than Provenance 2 (µ_p2_ = 33.8 µm; *p* = .009), and marginally different from Provenance 3 (µ_p3_ = 32.8 µm; *p* = .0596) (Figure 2h; Table 1). Conforming to trends commonly found in the literature (e.g., Brodribb, Jordan, & Carpenter, 2013), our data show significant negative logarithmic relationships between stomatal density and stomatal pore length in the field and in the greenhouse, although this relationship depends on leaf surface.

## 4 **Consistent with patterns of local adaptation**,**genetic divergence in water‐regulatory traits are related to hydrological processes on the landscape**

**FIGURE 3:**
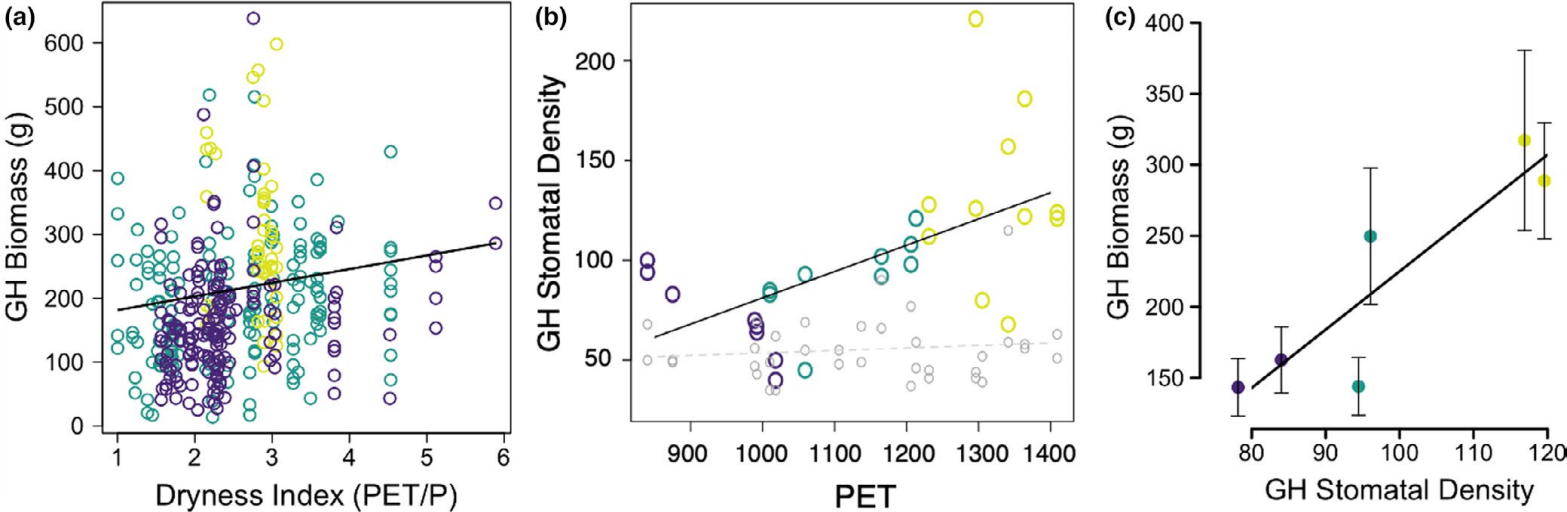
Water‐regulatory traits related to hydrologic variables on the landscape. (a) Greenhouse biomass (g) of *Populus angustifolia* increases with Dryness Index (PET/P) (estimate = 160.6, slope = 21.3, *N* = 381, *p* = .00025); (b) Greenhouse stomatal density increases with atmospheric demand (PET); Field stomatal density does not (gray dotted line and points); (c) Genetic Trait Correlation. Population means and standard errors of greenhouse stomatal density and biomass (R^2^ = 0.80, *p* = .016). Provenance color follows Figure 1 for all panels

In the common environment, we show that plant biomass is positively related to the dryness index (PET/P) with plants originating from more arid sites showing ~21.3 g more biomass for each unit on the dryness index (Figure 3a; *N* = 381, *p* = .00025**)**. In the common environment, the stomatal density (stomates/area) of *P. angustifolia* leaves increases as the atmospheric demand for water (PET) increases at plant site of origin (Figure 3b), although leaves in the field show no significant difference in stomatal density across this gradient (gray line; Figure 3b). Furthermore, higher biomass plants generally have higher water demands that may be reflected in stomatal density. We show that stomatal density is positively correlated to biomass (g) in greenhouse plants, accounting for 80% of the variation (Figure 3c **;** *R* ^2^ = .80, *p* = .016). In the field, stomatal ratio appears to be positively related to biomass (kg) of field plants (*R* ^2^ = .55, *p* = .089).

## 5 Water stable isotope compositions

**FIGURE 4:**
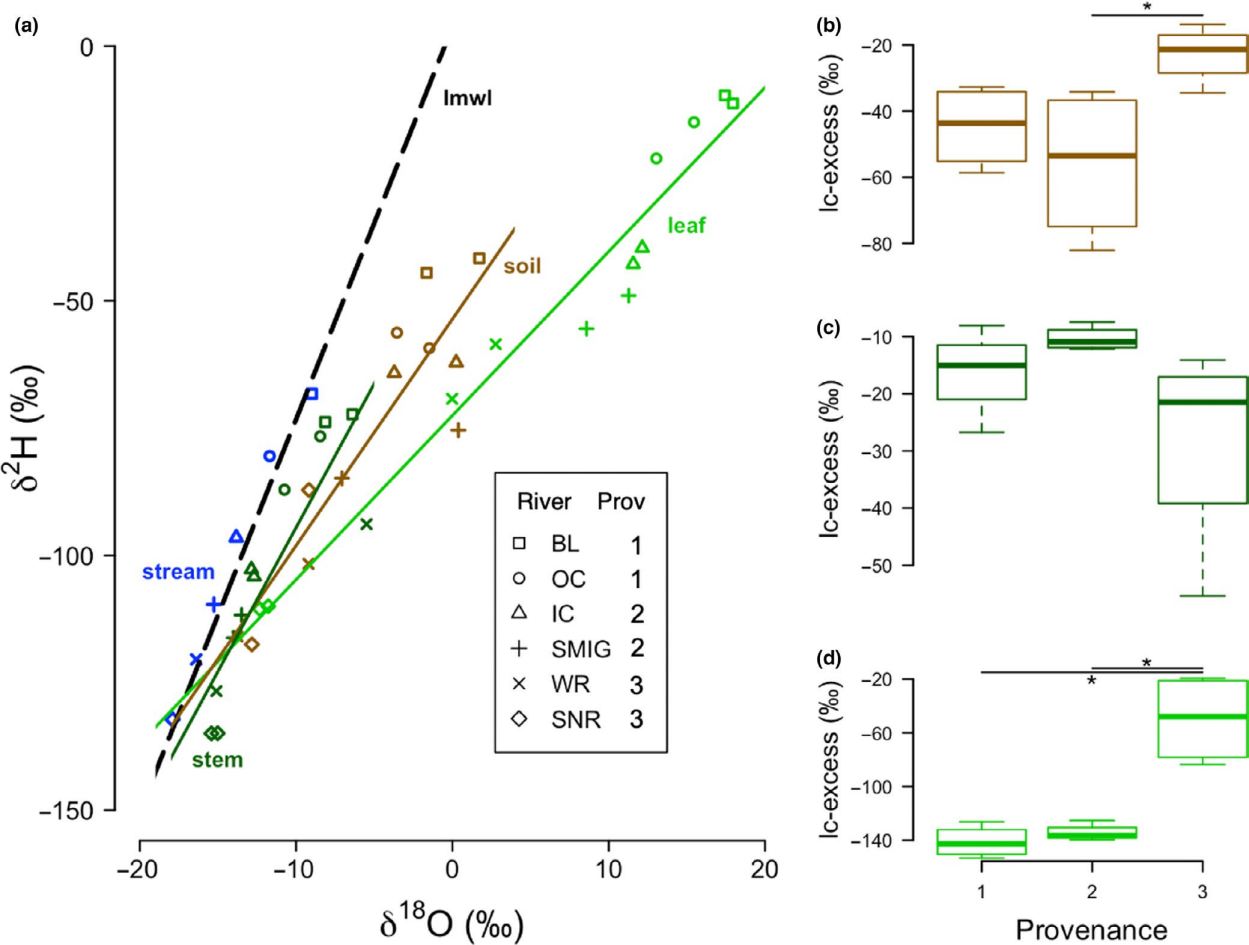
(a) Dual isotope plot comparing stable oxygen (^18^O/^16^O) and hydrogen (^2^H/^1^H) ratios (expressed per mille). The local meteoric water line (LMWL) representing isotopic composition of precipitation in June is represented by the dotted black line (y = 7.01x + 3.77, R^2^ = 0.99). Blue points represent stream samples collected at the mid‐elevation site along each of the six rivers. Soil (brown line; y = 4.45x – 53.6, R^2^ = 0.82), *P. angustifolia* stem (dark green; y = 5.65x – 38.07, R^2^ = 0.77) and leaf (light green; y = 3.22x −72.52, R^2^ = 0.95) samples are also represented. Populations are represented by symbols. Line conditioned excess values for soil (b), stem (c), and leaf (d) samples by provenance. Asterisks refer to statistically significant differences between provenances

Fitting expectations, our streamwater samples overlap the local meteoric water line (LMWL; Figure 4a; Table 2), and the stable isotope compositions from nonsaturated soil zones plot below the LMWL (Sprenger et al., 2016; Figure 4a). Negative lc‐excess values for soil samples indicate that the water in the nonsaturated soil zone was exposed to evaporative enrichment, and even more so in Provenances 1 and 2 (Figure 4b; Landwehr & Coplen, 2014; Sprenger et al., 2016). Also adhering to expectations, we find that lc‐excess in the soil is significantly correlated with streamflow in the month prior to collection (May, *R* ^2^ = .38, *p* = .033) and marginally correlated with mean annual streamflow (*R* ^2^ = .297, *p* = .066). Although we do not have deep groundwater samples for our sampling locations, groundwater is known to consistently plot along the LMWL (Sprenger et al., 2016). We acknowledge that throughfall water may already be enriched when it reaches the nonsaturated soil zone and that tree cover may decrease fractionation processes in soil (Sprenger et al., 2016). These stable isotope ratios (Figure 4) combined with mean annual values on the Budyko Curve (Figure 1b) which show higher atmospheric demand for water than the supply of it (PET/*p* > 1) confirm previous observations that riparian cottonwood forests do not get enough precipitation during the growing season to support the levels of transpiration to meet atmospheric demand (Flanagan et al. 2017, Yang et al., 2019).

**TABLE 2:** Summary of linear regression parameters for the relationship between oxygen (δ^18^O) and hydrogen isotopes (δ^2^H). Parameters are represented for precipitation (lmwl), stream, soil, stem, and leaf samples

Sample	Slope	Intercept	*r* ^2^
Imwl	7.01	3.77	0.988
Steam	7.39	2.30	0.983
Soil	4.45	−53.6	0.824
Stem	5.65	−38.1	0.771
Leaf	3.22	−72.5	0.954

**FIGURE 5:**
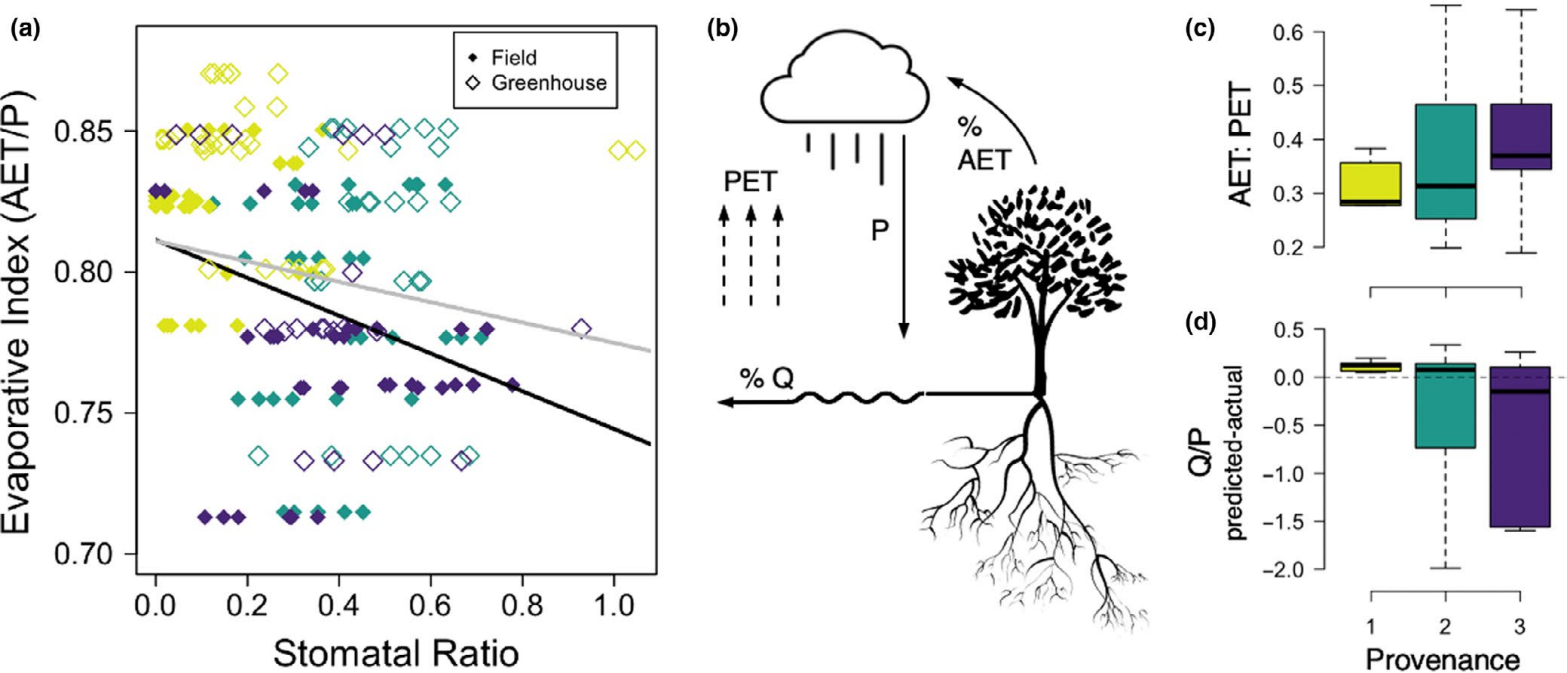
Landscape–Vegetation–Atmospheric Feedback. (a) The evaporative Index (AET/P) is significantly related to stomatal ratio in the field (filled diamonds, black line; R^2^= −0.35, *df* = 106, *p* = .00023) and in the greenhouse (open diamonds, grey line; R^2^= −0.23, *df* = 77, *p* = .039); (b) Conceptual figure representing role of vegetation in the water cycle; (c) The ratio of actual evapotranspiration (AET) to potential evapotranspiration (PET) indicates the relationship between water use on the landscape and atmospheric demand for water. The higher the ratio, the closer the landscape is to meeting the atmospheric demand for water; (d) Difference between predicted and actual streamflow, standardized by precipitation. Predicted streamflow standardized by precipitation was calculated as 1‐[AET/P]

*P. angustifolia* stem and leaf oxygen and hydrogen isotope compositions are shown in relation to the LMWL (precipitation), stream, and nonsaturated soil in Figure 4a. A linear regression between the stem water isotopes of hydrogen and oxygen has a lower slope than the LMWL suggesting that the water had been evaporatively enriched upon plant use (Figure 4a, Table 2). Although this regression is significant and shows good fit (Table 2; *R* ^2^ = .77), regressions of stem isotope compositions split by provenance each show a stronger fit (respectively by provenance, *R* ^2^ = .90, .95, .96), and different slopes (respectively by provenance, 3.5, 10.4, 3.9; Figure S2). Despite this, lc‐excess values of stem water do not significantly differ between provenances (Figure 4c). As expected, the slope for leaf isotopic composition is lower than all others, as leaves experience substantial isotopic enrichment during evapotranspiration (Figure 4a, Table 2 **)**. Values of lc‐excess in leaves are significantly higher in Provenance 3 samples (Figure 4d), supporting local adaptation patterns found in leaf traits (Figure 2) as well as the relationship between AET and stomatal ratio (Figure 5a). Stomatal ratio in the field is significantly correlated with leaf lc‐excess (*R* ^2^ = .53, *p* = .0076). Deuterium‐excess (d‐excess) values showed the same patterns as line conditioned excess values (lc‐excess).

Our results suggest that *P. angustifolia* populations may draw water from different water sources (stream, soil, or precipitation) and/or may have locally adapted rooting structures as lower lc‐excess values often correlate with shallower soil‐water use (Sprenger et al., 2016). Previous research on Populus in Arizona shows that some trees can opportunistically use precipitation water when it is available but rely on groundwater or streamwater during dry periods (Snyder & Williams, 2000). We acknowledge that drawing comparative inferences from soil and plant stable isotope data across space is cautioned (Goldsmith et al., 2019), as is assuming plant accession to specific water sources based on matching isotope compositions (Zhao et al., 2016).

## 6 Populations’ role in the water cycle varies on the landscape

The evaporative index (AET/P) represents the percentage of precipitation water recycled to the atmosphere through plants. Stomatal ratio, in field and greenhouse plants, is related to the evaporative index (AET/P) such that stomatal ratios are lower (more stomates on the bottom of leaves) when a higher percentage of available water is cycled back to the atmosphere in a given location (Figure 5a **)**. Although this relationship holds in both the field and the greenhouse, the relationship is~11.5% stronger in the field possibly indicating a level of trait plasticity (solid black line; Figure 5a). Despite a greater percentage of precipitation water being used by plants in Provenance 1 (EI; Figure 1b), the relationship between AET:PET (Figure 5c) shows that Provenance 1 is furthest from meeting atmospheric demand for water. Furthermore, with the Budyko model assumption that all water falling as precipitation is divided into AET or Q (streamflow), we predicted the fraction of water that should be available as streamflow across the landscapes where the three populations exist and compared that to actual streamflow data derived from the National Hydrology Dataset (NHDPlusV2; McKay, Bondelid, & Dewald, 2012). Predicted Q/P minus Actual Q/P (Figure 5d) shows that Provenances 1, and 2 (on average), hold less water in Q than actually predicted to be in that pool (positive values), whereas Provenance 3 has more water held in Q than predicted (negative values). Positive values indicate that water is “lost” or held in another pool that is not captured by this model (e.g., in plant biomass) while negative values indicate that water is supplied to the system by means other than precipitation (e.g., snowmelt).

## 7 DISCUSSION

### 7.1 Interacting global change gradients

Atmospheric hydrologic model parameters that capture variation in water‐energy interactions across landscapes do a 30% better job of explaining patterns of plant biomass than temperature and precipitation in statistical models (Figure S3), consistent with predictions that, although derived from temperature, PET should select more strongly than temperature on water‐use traits and plant biomass (Siepielski et al., 2017; Wright et al., 2004). While impossible to simultaneously consider all interacting gradients across a landscape, these hydrologic variables do capture nuances in climatic interactions that independent gradients of temperature and precipitation do not: For example, physical water‐energy interactions on the landscape vary across factors such as soil type, vegetation type and cover, and other biotic factors that the metrics in this study inherently capture (Ambrose & Sterling, 2014; Brown, Gillooly, Allen, Savage, & West, 2004; Troch et al., 2013; Zhang, Dawes, & Walker, 2001). Temperature and water on the landscape are fundamental regulators of plant growth, survival, and reproduction (Guisan & Zimmermann, 2000) and thus are critical to the functioning and persistence of ecosystems. A 2016 review of plant distribution models revealed that temperature and water‐related variables appear in 88.5% of models, but that water‐related variables that *depend* on temperature (e.g., evapotranspiration, moisture deficit) appeared in <20% of the models (Mod, Scherrer, Luoto, & Guisan, 2016). Our results and this identified gap in modeling distributions highlight the importance of including variables that more accurately represent the availability of water in ecosystems and demands for water from the atmosphere. Understanding complex interactions of global change gradients is a significant challenge for modeling the evolutionary (e.g., plant adaptation) and ecosystem consequences (e.g., plant function) of climate change.

### 7.2 Evolution

Variation in water‐use traits will determine plant response to changing water availability on the landscape. We show that stomatal traits and plant biomass have evolved among genetic groups of *P. angustifolia* across a landscape gradient of dryness (PET/P). Plants derived from more arid regions (higher dryness index values) produced more biomass in the greenhouse and biomass was positively related to stomatal density (Figure 3c). These results conform to those found previously in *P. trichocarpa*, *P. balsamifera*,*and P. angustifolia* (Guy & Gornall, 2007; Soolanayakanahally et al., 2009). Interestingly, Kaluthota et al., 2015 found that differences in density between provenances were not related to aridity (Kaluthota et al., 2015) confusing the relationship we found that supports predictions that plants with high stomatal conductance in dry conditions may demonstrate rapid opportunistic biomass production (rate of photosynthesis) during infrequent or short periods of water availability (Hetherington & Woodward, 2003; Snyder & Williams, 2000). Conversely, populations derived from regions with historically high water supply may be less able to control water use and be at higher risk to drought‐induced mortality (Dudley, 1996), although experiments are necessary to confirm these predictions (e.g., Barton, Jones, Edwards, Shiels, & Knight, 2020). Numerous other physiological studies on *Populus* species show that water stress through reductions in precipitation, groundwater, or streamflow, can lower leaf gas exchange, water potentials, xylem cavitation, stomatal conductance, and net photosynthetic rates (Horton, Kolb, & Hart, 2001; Rood et al., 2003; Tyree, Kolb, Rood, & Patino, 1994), resulting in morphological changes such as lower biomass production, increased branch sacrifice and crown reduction, leaf size, or stomatal size and number (Dunlap & Stettler, 2001; Rood et al., 2003; Rood, Patiño, Coombs, & Tyree, 2000). On the other hand, inundation with water, as would occur with flooding, has been shown to lower net photosynthetic rate, stomatal conductance, transpiration, and growth in *Populus* (Amlin & Rood, 2001; Rood et al., 2010). Varying responses to these two extremes of water stress, drought, and flooding, emphasize the need to consider population‐level responses to multiple aspects of the water cycle.

Global variation in plant growth is predominantly attributed to temperature and water (Babst et al., 2019; Bates et al., 2008; Jones et al., 2012; Lytle & Poff, 2004; Milly, Dunne, & Vecchia, 2005; Poff & Zimmerman, 2010). As temperature increases, trees are becoming increasingly limited by water as the atmospheric demand for water (PET) increases (Babst et al., 2019; Novick et al., 2016). In relation to landscape water supply and demand, we show biomass and stomatal traits differ between field and greenhouse trees, suggesting that plasticity in these correlated traits may also vary on the landscape. Although, whether there are genetically based differences in phenotypic plasticity requires further study (e.g., Barton et al., 2020) of population tolerance to environmental conditions as well as their capacity to display a range of phenotypes (Nicotra et al., 2010). If plastic, variation in these traits could affect population responses to a changing climate—either buffering against rapid environmental change or assisting in adaptation (Chevin, Lande, & Mace, 2010; Lande, 2009; Nicotra et al., 2010); could modify the strength and direction of plant–atmosphere feedbacks.

### 7.3 Feedback

Much variation in ecosystem function depends on the metabolic—often adaptive—characteristics of individual organisms, which are governed by laws of mass and energy balance (Brown et al., 2004). Above, we discussed how large‐scale mass‐energy relationships of the water cycle drive the evolution of plant populations to control water use (Figure 3a,b). These trait differences surely manifest in the observed landscape patterns seen in: (a) actual evapotranspiration (AET) on the landscape (Figure 5a); (b) the relationship between AET and PET on the landscape (Figure 5c); and, (c) predictions of Q on the landscape (Figure 5d). Transpiration totals, on average, 80%‐90% of evapotranspiration on the landscape (Jasechko et al., 2013), such that these genetically based trait divergences across plant populations (Figure 2) should cause populations to respond, and feedback, differently to water and energy availability. Because the water cycle is influenced significantly by genetically based plant traits, we demonstrate how among‐population level evolutionary processes can result in variation in plant–atmosphere feedbacks on a geographic scale. All other work at this scale has been in the context of plant–soil relationships (Senior et al., 2018; Van Nuland et al., 2016, 2017; Van Nuland, Ware, Bailey, & Schweitzer, 2019; Ware, Fitzpatrick, et al., 2019). In drought conditions, the ability of plants to control water can alter feedbacks to the atmosphere (AET), while the ability of plants to opportunistically obtain water from different sources (e.g., Snyder & Williams, 2000) may alter stream flow (Q) and entire stream ecosystems.

Scaling ecosystem feedbacks to global processes is a difficult challenge for ecosystem ecologists yet is crucial for understanding how populations are spatially distributed and the selective forces that act on the populations. Functional traits of organisms generally vary across large environmental gradients making it likely that similar feedbacks are common due to the interaction between environmental gradients and legacy effects of trait‐based species interactions (Van Nuland et al., 2019; Ware, Van Nuland, et al., 2019; Ware, Fitzpatrick, et al., 2019). A separate exploration of the Budyko model revealed plant adaptations to be simultaneously a cause and consequence of the water cycle, showing how rooting structure and transpiration efficiency have adapted to the dryness index (Gentine et al., 2012)—plant adaptations can profoundly control the annual water cycle, revealing mechanisms for eco‐evolutionary feedbacks (Eagleson, 1978; Eagleson & Tellers, 1982; Gentine et al., 2012). Similarly, soil moisture in zones of hybrid *Populus* (cross between parent species *P. angustifolia* and *P. fremontii*) was found to be lower than in adjacent zones dominated by the parent species (Schweitzer, Martinsen, & Whitham, 2002), reinforcing that genetically based differences in transpiration rates (Fischer, Hart, Whitham, Martinsen, & Keim, 2004) and water‐use traits (shown here) can be the basis for discovering feedbacks between population genetic variation and long‐term variation in ecosystem fluxes of energy and water across large landscapes.

### 7.4 Implications

Increased drought conditions are predicted to become more widespread and more severe in many geographic locations (Famiglietti, 2014; Georgakakos et al., 2014; Milly et al., 2005). The western United States is currently experiencing a 1000‐year drought threatening the most diverse ecosystems in the desert (riparian ecosystems) with widespread mortality (Gitlin et al., 2006; Kominoski et al., 2013). Occurring at the terrestrial–freshwater interface (Naiman & Décamps, 1997), riparian ecosystems are likely to be affected by changes to many aspects of the water cycle, such as streamflow or the atmospheric demand for water, as well as precipitation (Lytle & Poff, 2004; Milly et al., 2005; Perry, Andersen, Reynolds, Nelson, & Shafroth, 2012; Poff & Zimmerman, 2010; Rood et al., 2003). Although threatened, these systems may be “hotspots” for adaptation to climate change as they historically have been highly exposed to extremes of these various climatic stimuli (Capon et al., 2013). We demonstrated that biomass and stomatal traits, estimates of carbon acquisition, primary productivity, and water‐use efficiency (Cornelissen et al., 2003), differ across populations of an foundational riparian tree. These adaptations are important for the plant and the entire ecosystem to deal with drought (Aasamaa et al., 2001; Cornelissen et al., 2003; Hetherington & Woodward, 2003; Sack et al., 2006). In drought conditions, the ability of plants to control water may alter feedbacks to the atmosphere (AET; Figure 5a‐c), while the ability of plants to obtain water from different sources may alter stream flow (Q; Figure 5b,d) and the greater stream ecosystem.

### 7.5 Final conclusions

Integrating ecohydrology and landscape‐level genetic variation using the theoretical Budyko Curve allowed us to consider fluxes of energy and matter, interacting climatic gradients, and population genetic structure together to understand linkages between large‐scale hydrologic processes and evolutionary processes. The model accounts for interactions between temperature and water which enact long‐term selection pressures on plant traits and captures the key role plants play in the ecosystem through recycling water to the atmosphere. Combined, results indicate a landscape‐scale feedback and provide information about where populations and watersheds may be at risk and where ecosystem processes may be stable.

## CONFLICT OF INTEREST

The authors declare no conflict of interest.

[Correction added on 11 June 2021, after first online publication: Conflict of Interest statement added to provide full transparency.]

## AUTHORS' CONTRIBUTIONS

SLJB and JKB conceived of the manuscript. SLJB collected data, analyzed data, and wrote manuscript. LOM, IMW, JAS, and JKB all assisted with data collection and provided significant editorial and analytical advice.

## Supporting information

Fig S1

Fig S2

Fig S3
