# Supplementary figures and images for: Plant genetic variation drives geographic differences in atmosphere–plant–ecosystem feedbacks
Source: Plant Environ Interact. 2020 Sep 28;1(3):166–80. doi: 10.1002/pei3.10031 (PMC10168077; doi:10.1002/pei3.10031)

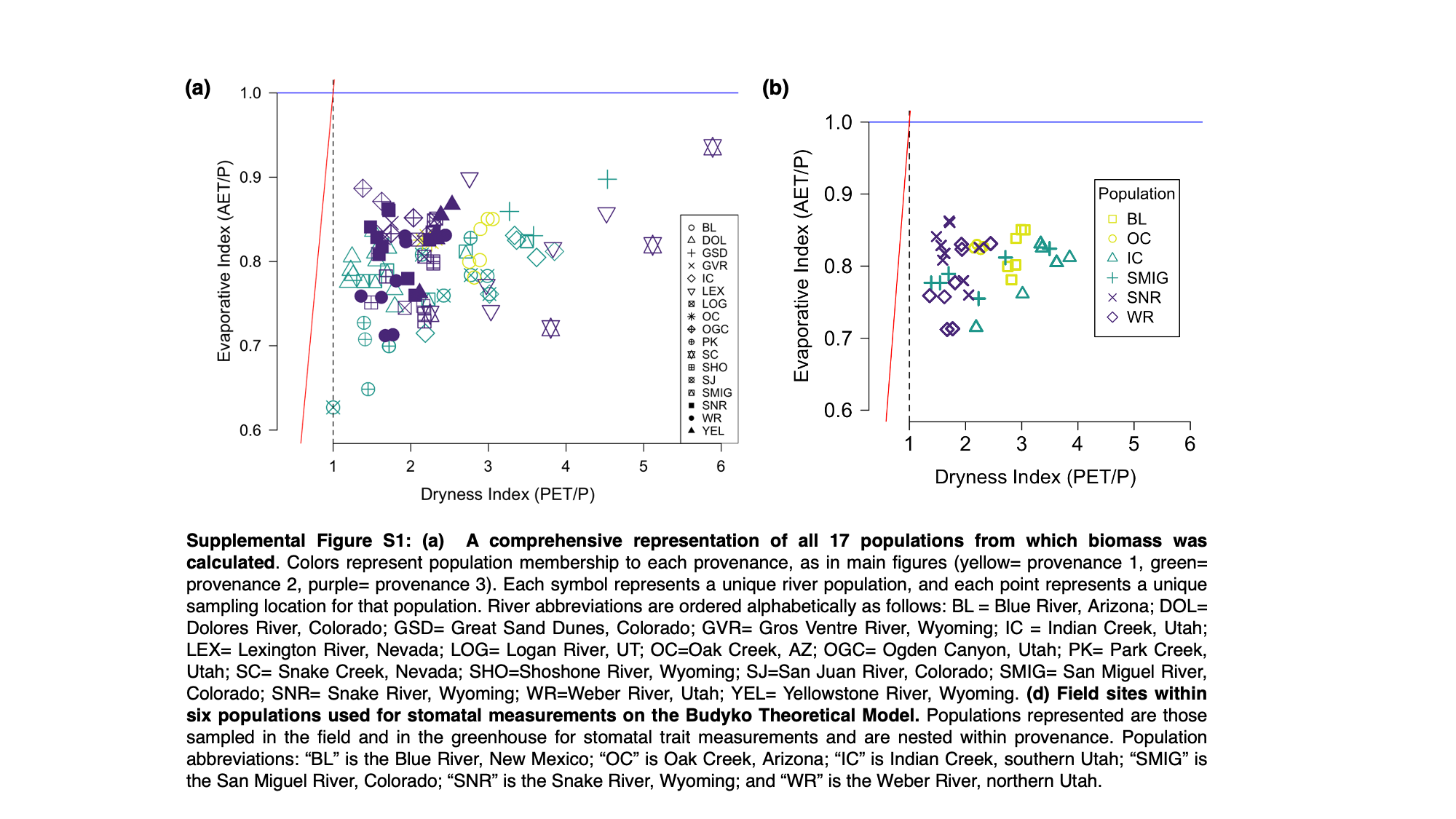

Supplement: Supplementary file 1 — Fig S1 [file PEI3-1-166-s002.tiff]

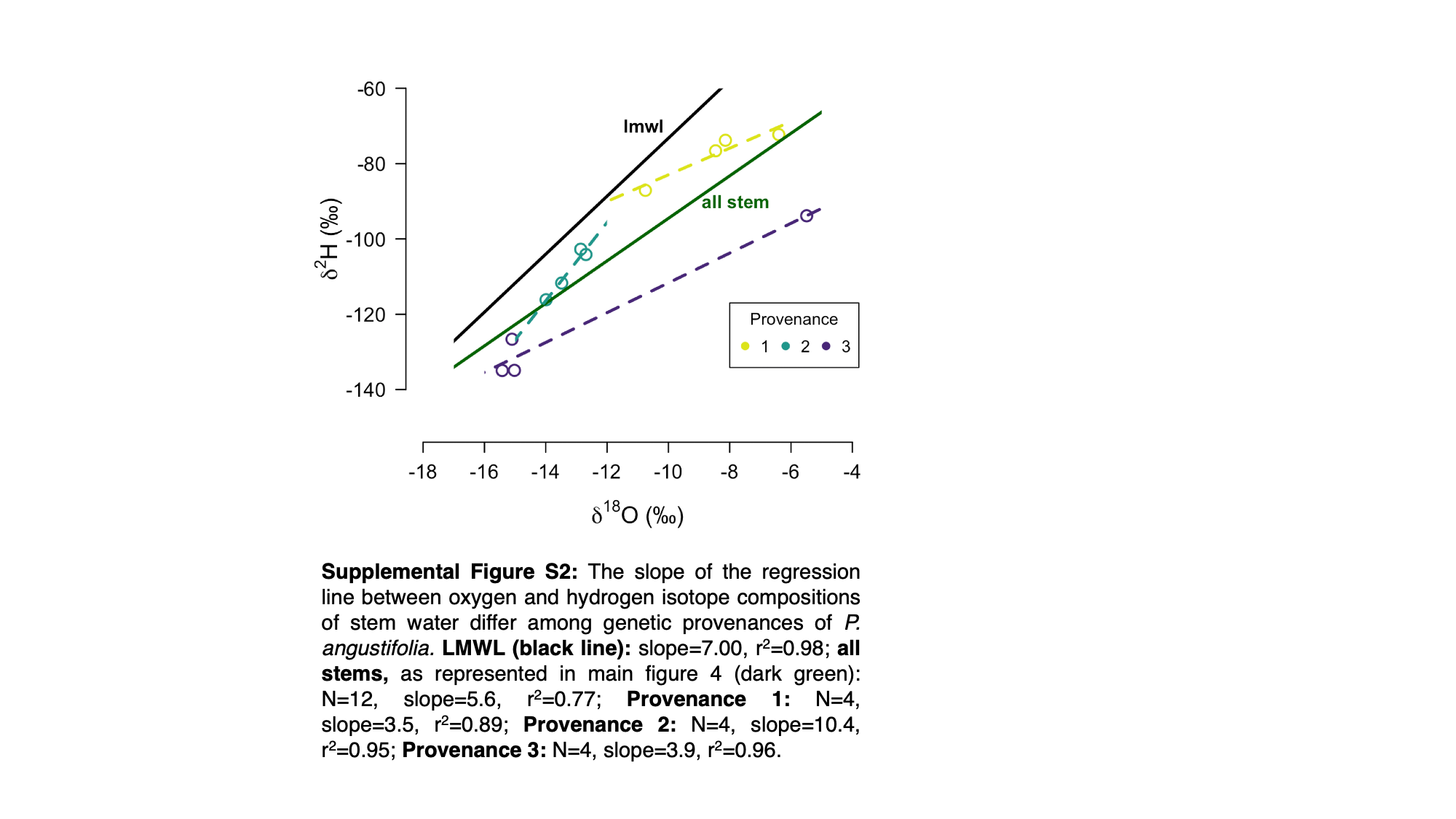

Supplement: Supplementary file 2 — Fig S2 [file PEI3-1-166-s001.tiff]

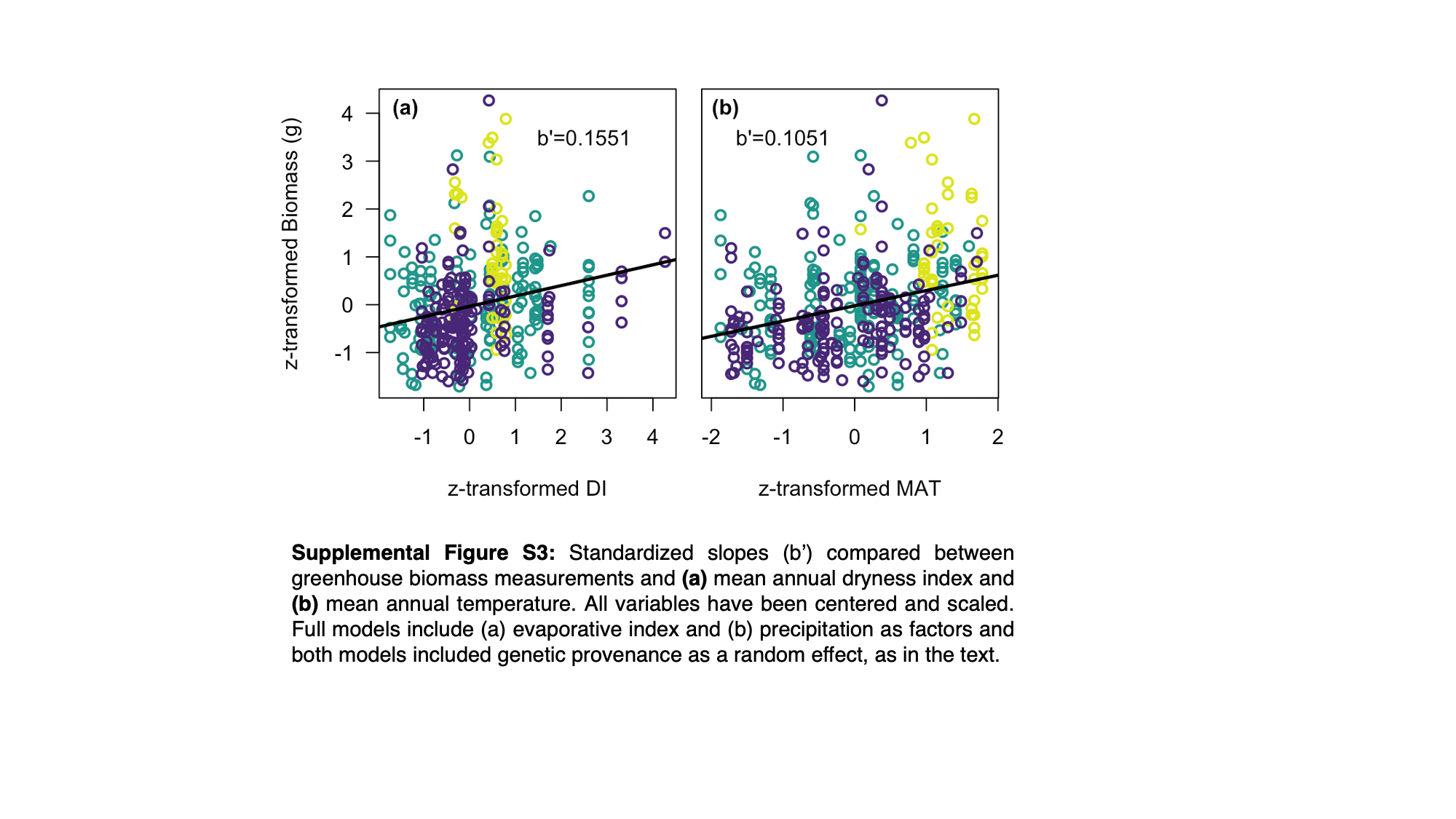

Supplement: Supplementary file 3 — Fig S3 [file PEI3-1-166-s003.tiff]
